# Dynamics of fully nonlinear capillary–gravity solitary waves under normal electric fields

**DOI:** 10.1007/s10665-017-9912-z

**Published:** 2017-06-09

**Authors:** T. Gao, P. A. Milewski, D. T. Papageorgiou, J.-M. Vanden-Broeck

**Affiliations:** 1 Department of Mathematics, Imperial College London, London, SW7 2AZ UK; 2 Department of Mathematics, University College London, London, WC1E 6BT UK; 3 Department of Mathematical Sciences, University of Bath, Bath, BA2 7AY UK

**Keywords:** Surface wave, Solitary wave, Wave interactions

## Abstract

Two-dimensional capillary–gravity waves travelling under the effect of a vertical electric field are considered. The fluid is assumed to be a dielectric of infinite depth. It is bounded above by another fluid which is hydrodynamically passive and perfectly conducting. The problem is solved numerically by time-dependent conformal mapping methods. Fully nonlinear waves are presented, and their stability and dynamics are studied.

## Introduction

Water waves propagating on the interface between two fluids have been studied intensively using either analytical or numerical methods. Many different mathematical methods have been introduced to study the steady or time-dependent solutions both in shallow and deep waters (for review, see, e.g. [1, 2] and the references therein). In the case of deep water, it is well acknowledged that there exist two families of capillary–gravity solitary waves bifurcating from the minimum of phase speed-denoted elevation and depression waves. In [3], the stability of these waves was studied using a numerical spectral analysis. It was found that depression waves with single-valued profiles were stable, whereas there was a stability exchange on the branch of elevation waves. These results were later verified numerically by Milewski et al. [1]. Recently, the problem of dynamics and stability was investigated systematically by Wang [4] where the depression waves with overhanging structure were proved to be also stable. On the experimental side, early experiments on three-dimensional capillary–gravity waves in a wind-wave tank were carried out by Zhang [5]. Fully localised lumps were observed. Later wavepacket solitary waves were generated in deep water by Diorio et al. [6].

In the presence of electric fields, this topic attracted much attention because it has many physical and industrial applications such as cooling systems and coating processes. In [7, 8], capillary waves on a fluid sheet under the effects of horizontal electric fields were studied. Fully nonlinear solutions were computed using a boundary integral equation method, and weakly nonlinear solutions were studied analytically by assuming the long-wave approximation limit. The effect of vertical electric fields was investigated in [9–11] where an asymptotic model equation for long waves was derived. Once again fully nonlinear solutions were computed by a boundary integral equation method and compared to the ones produced by the long-wave equation. To our knowledge, there have been, so far, no studies of time-dependent fully nonlinear water waves under the influence of electric fields.

The present work considers a two-dimensional dielectric fluid of infinite depth bounded above by a perfectly conducting gas such as plasma. The electric field is applied vertically throughout the space. This particular configuration converts a two-layer problem to the one of a one layer. Therefore, all the mathematical techniques which are used to solve the capillary–gravity problem can be inherited. Future work will involve cases where the upper layer also plays an active role in the dynamics.

The paper is structured as follows. In Sect. 2, we formulate the problem mathematically. Linear and weakly nonlinear asymptotic analyses are performed as well. In Sect. 3, a numerical method based on a time-dependent conformal map is introduced. A variety of numerical solutions are shown in Sect. 4. Finally conclusions are presented in Sect. 5.

**Fig. 1:**
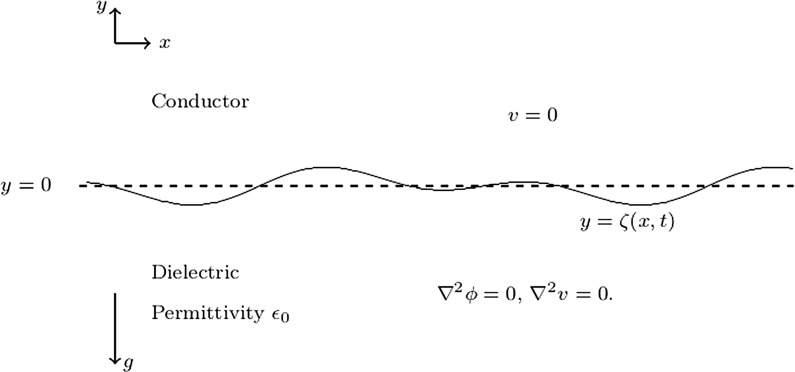
Configuration of the problem. The gravity acts in the negative *y*-direction. We denote the equation of the unknown free surface by $y=\zeta (x,t)$

## Formulation

We consider the two-dimensional irrotational flow of an inviscid incompressible fluid of infinite depth, which is bounded above by a hydrodynamically passive region also of infinite extent vertically. The fluid is assumed to be a perfect dielectric with permittivity $\epsilon _0$. The passive region above the fluid is assumed to be perfectly conducting. The problem can be formulated by means of cartesian coordinates with the *y*-axis directed vertically upwards and $y=0$ at the undisturbed level (Fig. 1). The gravity *g* and the surface tension $\sigma$ are both included in the formulation. The deformation of the free surface is denoted by $\zeta (x,t)$. A vertical electric field with voltage potential *v* is applied. We assume that $v \sim V_0y$ as $y\rightarrow -\infty$, where $V_0$ is a constant. Since the fluid motion can be described by a velocity potential function $\phi (x,y,t)$, introducing dimensionless variables by choosing

1 $$\begin{aligned} \left( \frac{\sigma }{\rho g}\right) ^{\frac{1}{2}},\quad \left( \frac{\sigma }{\rho g^3}\right) ^{\frac{1}{4}},\quad V_0 \end{aligned}$$

as the reference length, time and voltage potential, the governing equations can then be written as

2 $$\begin{aligned}&\nabla ^2\phi =0, \qquad \qquad \quad \text {for}\quad y<\zeta (x,t), \end{aligned}$$

3 $$\begin{aligned}&\nabla ^2 v=0, \qquad \qquad \quad \text {for}\quad y<\zeta (x,t),\end{aligned}$$

4 $$\begin{aligned}&\zeta _t=\phi _y-\phi _x\zeta _x, \quad \; \text {on}\quad y=\zeta (x,t),\end{aligned}$$

5 $$\begin{aligned}&v=0, \qquad \qquad \qquad \; \text {on}\quad y=\zeta (x,t),\end{aligned}$$

6 $$\begin{aligned}&v_y\sim 1, \qquad \qquad \qquad \text {as}\quad y\rightarrow -\infty ,\end{aligned}$$

7 $$\begin{aligned}&\phi _y\rightarrow 0, \qquad \qquad \quad \; \text {as}\quad y\rightarrow -\infty . \end{aligned}$$

and

8 $$\begin{aligned} \phi _t= & {} -\frac{1}{2}|\nabla \phi |^2-y + \frac{\beta }{1+\zeta _x^2} \Big [\frac{1}{2}\big (1-\zeta _x^2\big )\big (v_x^2-v_y^2\big )+2\zeta _x v_xv_y\Big ] +\frac{\zeta _{xx}}{\big (1+\zeta _x^2\big )^{3/2}},\quad \text {on}\quad y=\zeta (x,t), \end{aligned}$$

where the subscripts denote partial derivatives and

9 $$\begin{aligned} \beta =\epsilon _0 V_0^2\sqrt{\rho g/\sigma ^3} \end{aligned}$$

is a parameter which measures the ratio of electric forces to gravitational or surface tension forces [the latter two are in balance by our scaling (1)]. The last three terms of (8) are the forces due to gravity, the Maxwell stresses due to the electric field and surface tension. Equations (4) and (7) are the kinematic boundary conditions and the zero flow at minus infinity condition. The condition (5) expresses the fact that the region above the fluid is a perfect conductor, and in turn implies

10 $$\begin{aligned} v_x+v_y\zeta _x=0,\quad \text {on}\quad y=\zeta (x,t). \end{aligned}$$

Using condition (10) allows us to manipulate the electric field term in the dynamic boundary condition (8) to find

11 $$\begin{aligned} \phi _t=-\frac{1}{2} |\nabla \phi |^2-y-\frac{\beta }{2}|\nabla v|^2+\frac{\zeta _{xx}}{\left( 1+\zeta _x^2\right) ^{3/2}}\quad \text {on}\quad y=\zeta (x,t). \end{aligned}$$

The Hamiltonian of the system is defined by

12 $$\begin{aligned} H =\frac{1}{2}\int _{\mathbb {R}}\int _{-\infty }^\zeta |\nabla \phi |^2 \mathrm{{d}}y \, \mathrm{{d}}x+\frac{1}{2}\int _{\mathbb {R}}\zeta ^2 \mathrm{{d}}x+\frac{\beta }{2}\int _{\mathbb {R}}\int _{-\infty }^\zeta |\nabla v|^2 \mathrm{{d}}y \, \mathrm{{d}}x+\int _{\mathbb {R}}\left( \sqrt{1+\zeta _x^2}-1\right) \,\mathrm{{d}}x. \end{aligned}$$

It reduces to the classical form of the Hamiltonian for capillary–gravity waves when $\beta =0$. We denote the velocity potential on the free surface by $\varphi (x,t) \equiv \phi (x,\zeta (x,t),t)$. The kinematic and dynamic boundary conditions can be written in the canonical variables $\varphi$ and $\zeta$ as (see [12])

13 $$\begin{aligned} \zeta _t=\frac{\delta H}{\delta \varphi },\qquad \varphi _t=-\frac{\delta H}{\delta \zeta } . \end{aligned}$$

According to Saffman [13], a stability exchange of periodic waves due to superharmonic perturbations can only occur at

14 $$\begin{aligned} \text {either}\qquad \frac{\partial H}{\partial c}=0,\qquad \text {or}\qquad \frac{\partial c}{\partial H}=0, \end{aligned}$$

where *c* is the phase velocity. Saffman’s work was based on the Hamiltonian formulation (see [12]) where only gravity is considered. It can be generalised to include surface tension and electric fields due to the Hamiltonian structure (12), (13). Since all the perturbations are superharmonic for solitary waves, this argument is particularly useful in our numerical studies of stability.

A normal form analysis can be carried out by substituting the following ansatz into the governing equations

15 $$\begin{aligned} \zeta&=\epsilon A(\chi ,T)e^{ikx-i\omega t}+\epsilon ^2 \zeta _1+\epsilon ^3 \zeta _2+\cdots +\text {c.c.}, \end{aligned}$$

16 $$\begin{aligned} \phi&=\epsilon B(\chi ,T)e^{ikx-i\omega t+|k|y}+\epsilon ^2 \phi _1+\epsilon ^3 \phi _2+\cdots +\text {c.c.}, \end{aligned}$$

where $\epsilon$ is a small parameter, $T=\epsilon ^2 t$, and $\chi =\epsilon (x-c_gt)$. Here $c_g$ is the group speed and c.c. denotes the complex conjugate. Details of the derivation can be found in [14–16]. Here we just present the results from the first three orders. At $O(\epsilon )$, we retrieve the linear dispersion relation

17 $$\begin{aligned} \omega ^2=|k|(1+k^2)-\beta k^2\qquad \text {or}\qquad c^2=\frac{1}{|k|}+|k|-\beta , \end{aligned}$$

where we have assumed that waves are travelling in the positive *x*-direction. The dispersion relation admits a minimum of *c* at $k=1$ whenever $0\le \beta <2$. This minimum phase speed is denoted by $c^*$. Wave-packet like solitary waves with decaying tails bifurcate from that point. We have immediately

18 $$\begin{aligned} c^*=\sqrt{2-\beta }. \end{aligned}$$

At $O(\epsilon ^2)$, we obtain the expression for the group speed

19 $$\begin{aligned} c_g=\frac{1}{2\omega }(1+3k^2-2\beta |k|). \end{aligned}$$

We proceed to the next order to get the cubic nonlinear Schrödinger equation (NLS)

20 $$\begin{aligned} iA_T+\lambda A_{\chi \chi }+\mu |A|^2A=0, \end{aligned}$$

where

21 $$\begin{aligned} \lambda =\frac{|k|}{2\omega }\qquad \text {and} \qquad \mu =\frac{|k|^3\left( k^4+12\beta |k|^3+\frac{1}{2} k^2-3\beta |k| +4\right) }{2\omega (2k^2-1)}. \end{aligned}$$

The bifurcation to solitary waves takes place at $k=1$, where the NLS reads

22 $$\begin{aligned} iA_T+ \frac{1}{2\sqrt{2-\beta }} A_{\chi \chi }+\frac{11+18\beta }{4\sqrt{2-\beta }} |A|^2A=0. \end{aligned}$$

The NLS is of focusing type for $0<\beta <2$. It predicts the existence of bright solitons the envelope *A* of which has the explicit solution:

23 $$\begin{aligned} A(\chi ,T)=\sqrt{\frac{2C}{\mu }}\mathrm{sech}\,\left( \sqrt{\frac{C}{\lambda }}\chi \right) \mathrm{e}^{iCT}, \end{aligned}$$

where *C* is a constant. If $\beta >2$, the electric field destabilises the system therefore no solitary waves can exist. When $\beta$ approaches 2, the coefficients of $A_{\chi \chi }$ and $|A|^2 A$ in (22) both tend to infinity. The variable *T* is required to be of order $O( {\sqrt{2-\beta }})$ to balance equation (22) which violates the initial assumptions when $\sqrt{2-\beta }=o(1)$. Consequently the amplitude and horizontal length need to be rescaled and for $\beta$ near 2 the envelope becomes very broad and small (we note that $\omega \rightarrow 0$ when $\beta \rightarrow 2$). It is expected that the solutions approach linear sinusoidal waves in the limit $\beta \rightarrow 2$.

## Numerical scheme

To find the unknown shape of the free surface, we use the time-dependent conformal mapping introduced in [17] which maps the free surface onto the horizontal axis in a new complex plane denoted by $(\xi ,\eta )$. The map can be formally defined as the solution of the following boundary value problem

24 $$\begin{aligned}&y_{\xi \xi }+y_{\eta \eta }=0, \qquad \text {for} \quad \eta <0,\end{aligned}$$

25 $$\begin{aligned}&y=Y(\xi ), \qquad \qquad \; \text {on} \quad \eta =0,\end{aligned}$$

26 $$\begin{aligned}&y_{\eta }\rightarrow 1, \qquad \qquad \quad \; \text {as}\quad \eta \rightarrow -\infty , \end{aligned}$$

where $Y(\xi )=\zeta (\xi ,0)$. The harmonic conjugate $x(\xi ,\eta )$ can be obtained via the Cauchy-Riemann equations for the analytic function $z(\xi ,\eta )=x(\xi ,\eta )+iy(\xi ,\eta )$. Similarly we can derive the harmonic conjugates of $\phi (\xi ,\eta )$ and $v(\xi ,\eta )$, denoted by $\psi (\xi ,\eta )$ and $w(\xi ,\eta )$ respectively. In the mapped plane, we defined the surface variables as $X(\xi ,t)\equiv x(\xi ,0,t)$, $Y(\xi ,t)\equiv y(\xi ,0,t)$, $\varPhi (\xi ,t)\equiv \phi (\xi ,0,t)$, $\varPsi (\xi ,t)\equiv \psi (\xi ,0,t)$, $V(\xi ,t)\equiv v(\xi ,0,t)$ and $W(\xi ,t)\equiv w(\xi ,0,t)$. It can be shown that

27 $$\begin{aligned} X_\xi&=1-\mathcal {H}[Y_\xi ], \end{aligned}$$

28 $$\begin{aligned} \varPsi _\xi&=\mathcal {H}[\varPhi _\xi ],\end{aligned}$$

29 $$\begin{aligned} W_\xi&=-1+\mathcal {H}[V_\xi ], \end{aligned}$$

where $\mathcal {H[.]}$ is the Hilbert transform operator which is defined as

30 $$\begin{aligned} \mathcal {H}[f](\xi )=\text {PV}\int \frac{f(\xi ')}{\xi '-\xi }\mathrm{{d}}\xi ', \end{aligned}$$

where *PV* demotes the principal value of the integral. We note that $V_\xi =0$ as *v* is identically zero everywhere on the free surface. Next we follow [1] to derive the time-evolution equations

31 $$\begin{aligned} Y_t&=Y_\xi \mathcal {H}\Big [\frac{\varPsi _\xi }{J}\Big ] -X_\xi \frac{\varPsi _\xi }{J}, \end{aligned}$$

32 $$\begin{aligned} \varPhi _t&=\frac{1}{2J}\big (\varPsi _\xi ^2-\varPhi _\xi ^2\big )-Y-\frac{\beta }{2J} +\frac{X_\xi Y_{\xi \xi }-Y_\xi X_{\xi \xi }}{J^{3/2}}+\varPhi _\xi \mathcal {H}\Big [\frac{\varPsi _\xi }{J}\Big ], \end{aligned}$$

where $J=X_\xi ^2+Y_\xi ^2$ is the Jacobian of the conformal map. In the dynamical computations, we employ the fourth order Runge-Kutta method to advance in time. The mesh size in space and the time-step are usually chosen as $d\xi$=0.05 and $dt =2.5\times 10 ^{-4}$ respectively.

For travelling waves, all functions depend on $x-ct$. After similar calculations as those presented in [1], we have

33 $$\begin{aligned} \varPsi =cY. \end{aligned}$$

Then the resulting governing equation becomes

34 $$\begin{aligned} \frac{1}{2}(c^2+\beta )\left( \frac{1}{J}-1\right) +Y-\frac{X_\xi Y_{\xi \xi }-Y_\xi X_{\xi \xi }}{J^{3/2}}=0. \end{aligned}$$

The Hamiltonian (12) can be rewritten in dimensionless variables as

35 $$\begin{aligned} H =\frac{c^2}{2}\int _{\mathbb {R}}Y_\xi \mathcal {H}[Y]\mathrm{{d}}\xi +\frac{1}{2}\int _{\mathbb {R}}Y^2X_\xi \mathrm{{d}}\xi +\int _{\mathbb {R}}\left( \sqrt{J}-X_\xi \right) \mathrm{{d}}\xi +\frac{\beta }{2}\int _{\mathbb {R}}Y_\xi \mathcal {H}[Y]\mathrm{{d}}\xi , \end{aligned}$$

where the terms appearing on the right hand side correspond to the kinetic energy, the potential energy due to gravity, surface tension and the electric field, respectively.

For a given wave profile with phase speed $c=c_1$ and electric parameter $\beta =\beta _1$, equation (34) has the same wave profile as its solution for a different value of $\beta =\beta _2$ if the corresponding phase speed $c_2$ satisfies

36 $$\begin{aligned} c_2^2+\beta _2=c_1^2+\beta _1. \end{aligned}$$

For example, a depression wave with $c=1.4$ for $\beta =0$ has exactly the same profile as that of a depression wave with $c=0.6$ for $\beta =1.6$. In addition, we deduce from (35) that $H$ is independent of $\beta$ for a fixed wave profile due to (36). Travelling waves of our system are related directly to capillary–gravity waves through this relation, by setting $\beta =0$. For example, the minimal speed of depression capillary–gravity solitary waves is about 0.9275 (see e.g [4]) where the wave profile has a point of contact with a trapped bubble. Applying (36) implies that the branch of depression waves can be continued down to $c=0$ without trapped bubbles, for any $2>\beta >0.9275^2\simeq 0.86$. For elevation waves, the complicated structure of the capillary–gravity bifurcation curve together with (36) implies that, with electric fields, isolated branches of waves will appear and disappear at certain values of $\beta$. This phenomenon will be discussed further below. Lastly, we note that the evolution equations (31),(32) do not have a simple transformation into their capillary–gravity counterparts.

We approximate the solitary waves by very long periodic waves and use the Fourier representation

37 $$\begin{aligned} Y(\xi )=\sum _{n=1}^N a_n \cos \left( \frac{n\pi \xi }{L}\right) +b_n \sin \left( \frac{n\pi \xi }{L}\right) , \end{aligned}$$

where the series is truncated after *N* terms. The coefficients $$a_n$$ and $$b_n$$ are the unknowns to be found. In particular for symmetric waves, we choose $$\xi =0$$ to be the axis of symmetry, i.e. the coefficients $$b_n$$ are zero. The wavelength 2*L* and the number of modes *N* are chosen to be sufficiently large so that the solutions do not change when *L* and *N* are further increased. We discretise the domain $$[-L,L)$$ in the mapped plane with a uniform mesh. The dynamic boundary condition (34) is satisfied on these grid points. The resultant system is solved by Newton’s method for the unknown Fourier coefficients. In most computations, we use 4096 or 6144 collocation points. The iterations are stopped when the $$l^\infty $$-norm of residual errors are less than $$10^{-10}$$. This numerical approach has been successfully used in [1, 4, 18] for capillary–gravity waves and in [19–21] for flexural–gravity waves.

## Numerical results

### Travelling waves

We start by computing fully localised steady solutions for different values of $\beta$. When $\beta =0$, our computations agree with the classical results for the capillary–gravity problem. The branch of depression waves is a simple monotonic curve whereas the branch of elevation waves has a complicated structure which snakes back and forth. The reader is referred to [22] for more details.

**Fig. 2:**
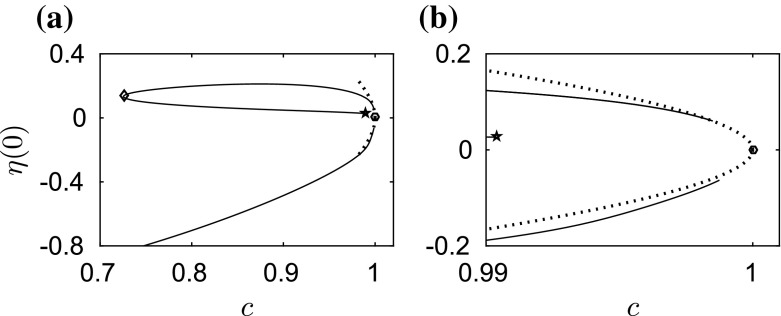
**a** Amplitude–speed bifurcation diagram (*solid curves*) of solitary waves for $\beta =1$. The first turning point with speed $c=0.7256$ is marked by a diamond. The second turning point with speed 0.9904 is marked by a pentagram. **b** A blow-up graph of (**a**) near the bifurcation point. The bifurcation points are marked as *circles*. The *dotted curves* are the asymptotic predictions of the NLS

**Fig. 3:**
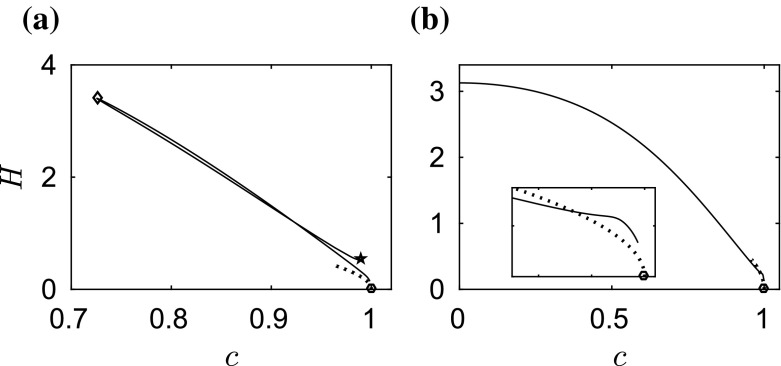
Energy–speed bifurcation diagram (*solid curves*) **a** for elevation waves and **b** for depression waves when $\beta =1$. The bifurcation points are marked as *circles*. The turning point in the *left* figure is marked by a *diamond*. The second turning point is marked by a *pentagram*. The *dotted curves* are the asymptotic predictions by the NLS

When $\beta =1$, we obtain qualitatively similar results to those of capillary–gravity waves. The amplitude–speed and energy–speed bifurcation diagrams are presented in Figs. 2 and 3, respectively, where depression (lower branch) and elevation (upper branch) solitary waves bifurcate from infinitesimal amplitude periodic waves at the minimum phase speed defined in (18). We only show part of the branch for better visualisation. The typical wave profiles are shown in Sect. 4.2 as the initial data of the time-dependent dynamics. The cubic NLS (20) can be used to predict the amplitude–speed and energy–speed relations near the bifurcation point (see e.g. [23, 24]) and these are superimposed as the dotted curves in Figs. 2 and 3. The bifurcation point of solitary waves approaches the origin as $\beta$ increases. This is particularly interesting for the branch of elevation waves, because its first turning point (marked by a diamond in Figs. 2 and 3) may touch the $c=0$ line for some critical value $\beta ^{\dagger }$, say. As (36) holds for different values of $\beta$ at the turning point of the branch of elevation waves, we evaluate $c^2+\beta$ for $\beta =1$ where the corresponding phase velocity is 0.7256 and for $\beta =\beta ^{\dagger }$ which is the limiting case with zero phase velocity. Then, we obtain

38 $$\begin{aligned} \beta ^{\dagger }=0.7256^2+1=1.5265. \end{aligned}$$

**Fig. 4:**
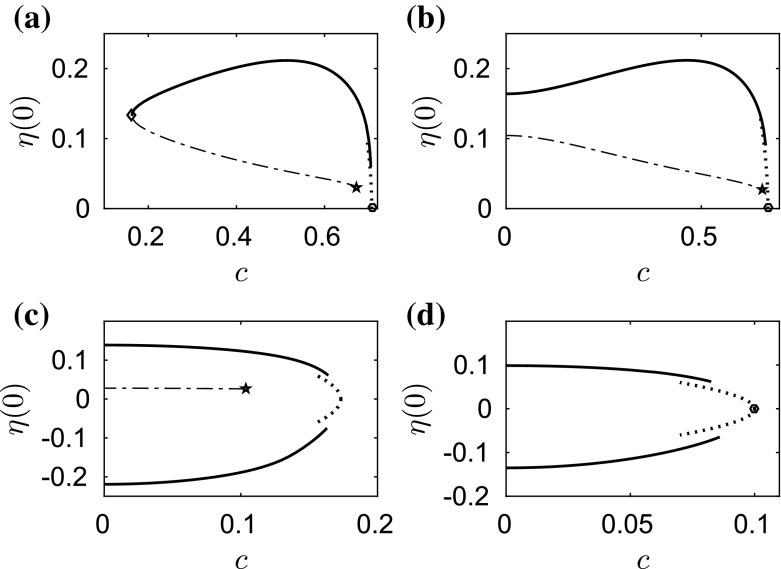
Amplitude–speed bifurcation diagram when **a** $\beta =1.5$, **b** $\beta =1.55$, **c** $\beta =1.97$ and **d** $\beta =1.99$. The depression waves are not presented for better display in (**a**) and (**b**). The *dashed curves* represent the lower part of the branch for elevation waves. The *dotted curves* are the asymptotic predictions of the NLS. The first turning point is marked by a *diamond* and the second turning point by a *pentagram*

We present two numerical examples for values of $\beta$ just smaller and larger than $\beta ^\dagger$, namely $\beta =1.5$ and $\beta =1.55$, as shown in Fig. 4a, b. It can be seen clearly that the turning point approaches $c=0$ line and touches it. When this happens, the branch of elevation waves split into two separate parts as shown in Fig. 4b. When the second turning point (marked by a pentagram in Figs. 2 and 3) touches the boundary $c=0$ at another critical value $\beta ^{\ddagger }$, the lower part of the branch for elevation waves disappears. The value of $\beta ^{\ddagger }$ can be estimated by using the same approach

39 $$\begin{aligned} \beta ^{\ddagger }=0.9904^2+1=1.9809. \end{aligned}$$

**Fig. 5:**
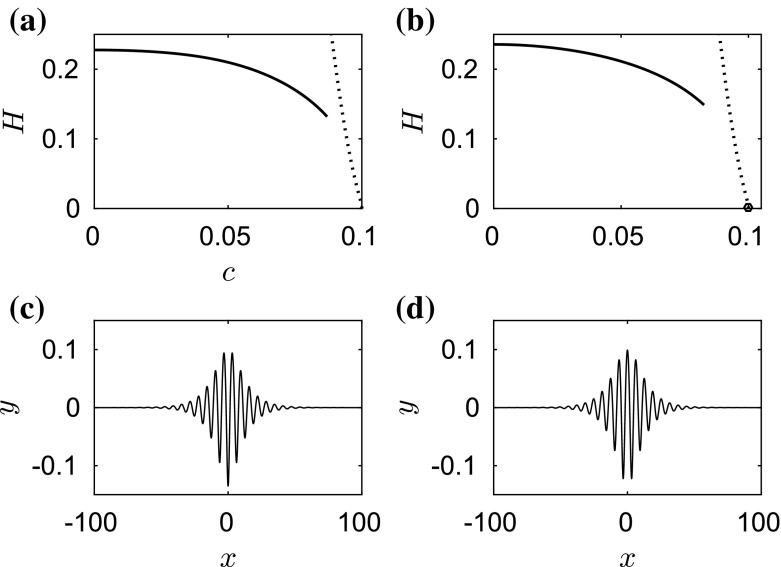
Energy–speed bifurcation diagram (*solid curves*) when $\beta =1.99$ for **a** depression waves, **b** elevation waves. The *dotted curves* are the predictions of the NLS. **c** The corresponding depression wave profile with wave speed $c=0$. **d** The corresponding elevation wave profile with wave speed $c=0$

We computed the bifurcation diagrams with $\beta =1.97$ (${<}\beta ^{\ddagger }$) and $\beta =1.99$ (${>}\beta ^{\ddagger }$) as shown in Fig. 4c, d. The lower part of the branch for elevation waves (dashed curve) disappears in this interval of $\beta$. The numerical results confirm (39). In particular when $\beta =1.99$, simple bifurcation diagrams for both depression and elevation waves have been obtained (see Fig. 4d). The two branches emanate from the bifurcation point and continue to $c=0$, where the corresponding wave profiles are plotted in Fig. 5c, d. In the energy–speed diagram shown in Fig. 2a, b, the branches are monotonic in the wave speed *c* and appear almost identical.

In conclusion, numerical evidence shows that the wave speeds are slowed down due to the effect of electric fields as predicted by (36). In addition, the branch of elevation waves split into two separate parts, when $\beta >\beta ^{\dagger }$, and the lower part disappears when $\beta >\beta ^{\ddagger }$. Our computations for $\beta =1$ stop at the pentagram in Fig. 2; however, the problem does not end there. The branch continues to snake back and forth where more turning points are expected. There exist other separate solution branches when $\beta >\beta ^{\dagger }$. As $\beta$ approaches 2 with many branches reaching $c=0$, there only remain two branches which are monotonic in *c*. In the next section, we investigate the stability of the computed solitary wave solutions with time-dependent computation.

### Stability analysis 

We now investigate numerically the stability of the solitary waves by introducing a small disturbance and solving the initial value problem. A frame of reference moving with the initial wave speed is always chosen, and we evolve the resultant initial condition in time. We recall that in the absence of the electric fields, the depression solitary waves are stable, whereas the elevation waves are unstable near the bifurcation point but become stable after the first turn of the branch of elevation waves at $c\simeq 1.24$ (see [1, 3, 4]).

**Fig. 6:**
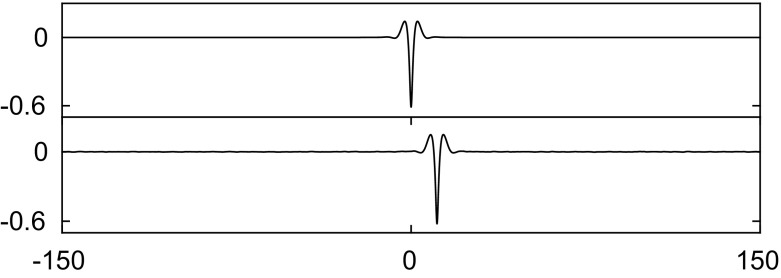
Numerical examination of the stability of a depression solitary wave with $c=0.83$ when $\beta =1$. The snapshots are taken at times: $t=0$ and 1000 (from *top* to *bottom*). The wave is initially perturbed by decreasing the amplitude by 5%. The profiles are sketched in the physical plane

We now describe our results with the inclusion of the electric fields. The numerical calculations presented here are carried out with $\beta =1$. The results are qualitatively similar to those of the capillary–gravity problem. A variety of perturbations with $\pm 5\%$ of the amplitude of the depression waves show no sign of instabilities. One example is presented in Fig. 6. A depression solitary wave ensues with smaller amplitude and therefore travelling faster than the original waves. This explains why the wave moves rightwards as time increases since the wave speed of the smaller wave is greater than the speed of the reference frame. As there is no stationary point or turning point on the curve of *H*(*c*) for depression waves, no stability exchange occurs on this branch, i.e. the depression branch is stable.

**Fig. 7:**
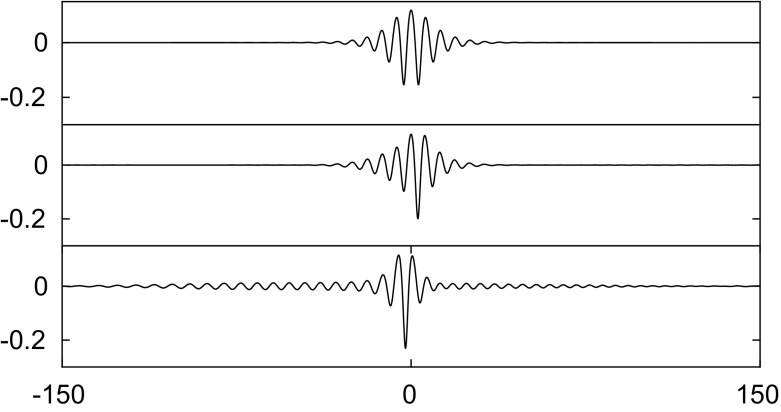
Numerical examination of the stability of an elevation solitary wave with $c=0.9916$ when $\beta =1$. The snapshots are taken at times: $t=0$, 400 and 1000 (from *top* to *bottom*). The wave is initially perturbed by $1\%$ of the amplitude. The profiles are sketched in the physical plane

**Fig. 8:**
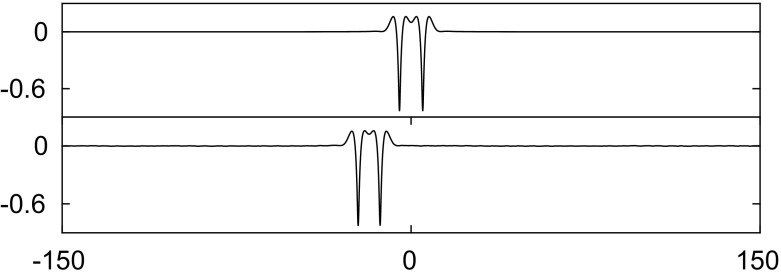
Numerical examination of the stability of an elevation solitary wave with $c=0.7547$ when $\beta =1$. The snapshots are taken at times: $t=0$ and 1000 (from *top* to *bottom*). The wave is initially perturbed with $+5\%$ of the amplitude. The profiles are sketched in the physical plane

Similarly, we apply a disturbance with $\pm 1\%$ of the amplitude to elevation waves from the elevation branch on the segment between the bifurcation point and the diamond symbol in Fig. 2. The wave eventually evolves into a depression solitary wave as presented in Fig. 7. On the next segment of elevation waves between the diamond and the pentagram, solitary waves are stable as confirmed by the numerical results in Fig. 8. Computations were carried out for other values of $\beta$ also and yielded similar results to those presented above.

**Fig. 9:**
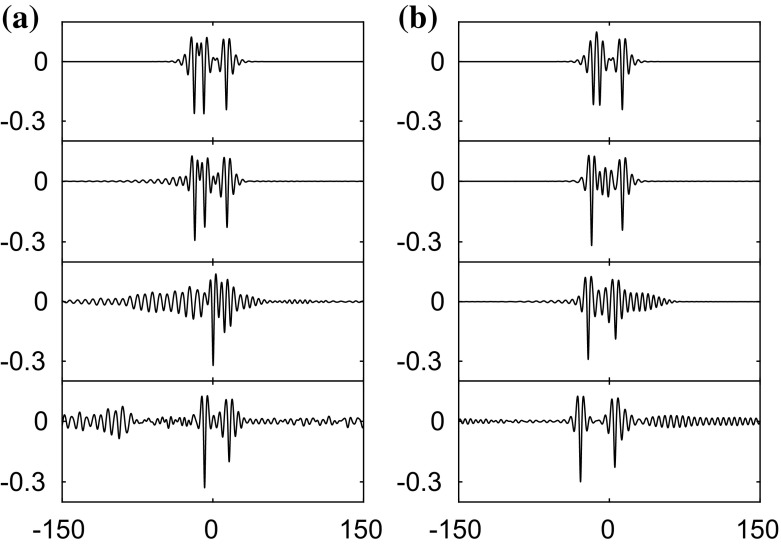
Numerical examination of the stability of two asymmetric solitary waves with $c=0.98$ when $\beta =1$. **a** The snapshots are taken at times: $t=0$, 1000, 1500 and 2000 (from *top* to *bottom*). **b** The snapshots are taken at times $t=0$, 250, 500 and 1000 (from *top* to *bottom*). The waves are initially perturbed by $-1\%$ of their amplitude. The profiles are sketched in the physical plane

Asymmetric solitary waves also exist in the presence of electric fields. Two examples are shown on the top left and top right in Fig. 9. By virtue of (36), the bifurcation structures of the solution branches for these two asymmetric solitary waves are guaranteed to be qualitatively similar to those presented in [4, 18]. In this work, we focus on the stability of these asymmetric waves. By adding an initial disturbance to these two asymmetric solitary waves, it is observed that both waves are unstable and evolve into two depression waves as confirmed in the figure. Similar numerical experiments in the absence of electric fields were carried out in [4].

### Collision

**Fig. 10:**
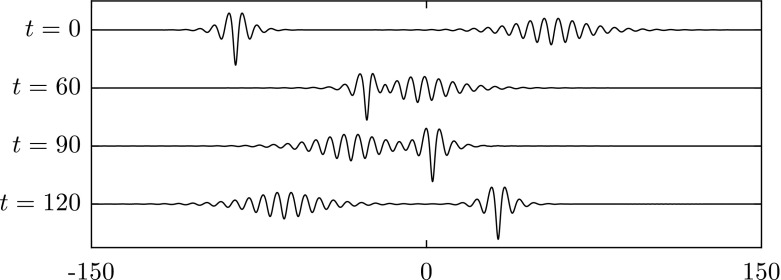
Head-on collision of two depression waves with $c=0.98$ (*left*) and $c=0.997$ (*right*) when $\beta =1$. Both waves survive after the interaction

**Fig. 11:**
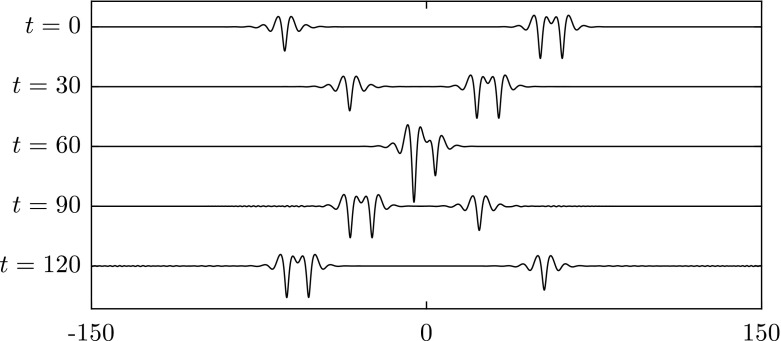
Head-on collision of a depression wave with $c=0.97$ (*left*), and an elevation wave with $c=0.9465$ (*right*) when $\beta =1$. Both waves survive after the interaction

In this section, we perform several numerical studies on wave interactions of stable solitary waves when $\beta =1$. The initial data are set up by superposing shifted solutions obtained in Sect. 4.1. The information on the velocity potential needed for the initial data can be retrieved by means of Eqs. (28) and (33). Head-on collisions are shown in Figs. 10 and 11; it is seen that both waves survive after the interaction and continue to travel with a slightly faster speed due to energy dispersion in the form of small ripples. These numerical solutions match the case of capillary–gravity waves studied in [1].

**Fig. 12:**
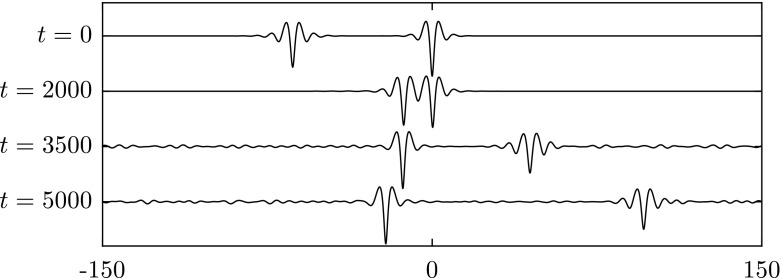
Overtaking collision of two depression waves $c=0.997$ (*left*) and $c=0.945$ (*right*) for $\beta =1$. A frame of reference moving with speed 0.945 is chosen. Both waves survive after the interaction

**Fig. 13:**
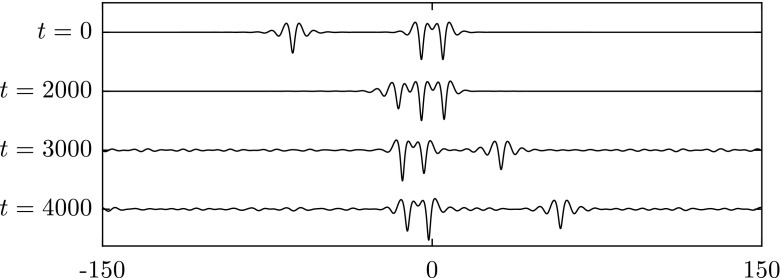
Overtaking collision of an elevation waves $c=0.97$ (*left*) and a depression wave with $c=0.945$ (*right*) for $\beta =1$. A frame of reference moving with speed 0.945 is chosen. Both waves survive after the collision

**Fig. 14:**
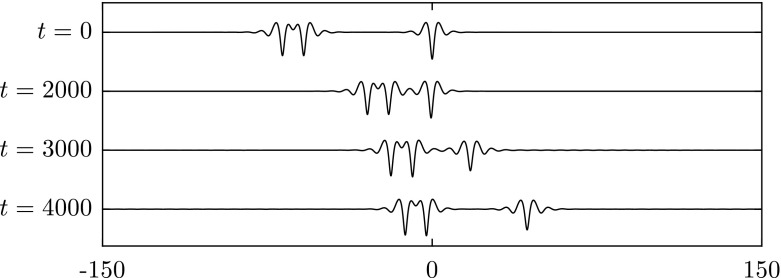
Overtaking collision of an elevation wave with $c=0.9641$ (*left*) and a depression wave with $c=0.945$ (*right*) when $\beta =1$. A frame of reference moving with speed 0.945 is chosen. Both waves survive and exchange speed after the collision

To study overtaking collisions, we place a solitary wave with a slower speed ahead of a faster solitary wave. A frame of reference is chosen to move with the slower wave. When the difference of two wave speeds is small, both waves survive and maintain their form. We have performed three numerical experiments as shown in Figs. 12,  13 and 14. In Fig. 12, a depression solitary wave overtakes another. Both waves survive, and there are small ripples generated after the collision. It is interesting to note from the results in Fig. 13 that the double-trough solitary wave emerges from the collision as a breather: a travelling localised wave with a periodic oscillation (note the troughs oscillate in amplitude at $t=3000,4000$). This emergence of breathers has been noted previously in [24]. In Fig. 14, the energy has been transferred from one wave to another during the interaction. As a result of this, the depression wave travels faster after the collision.

**Fig. 15:**
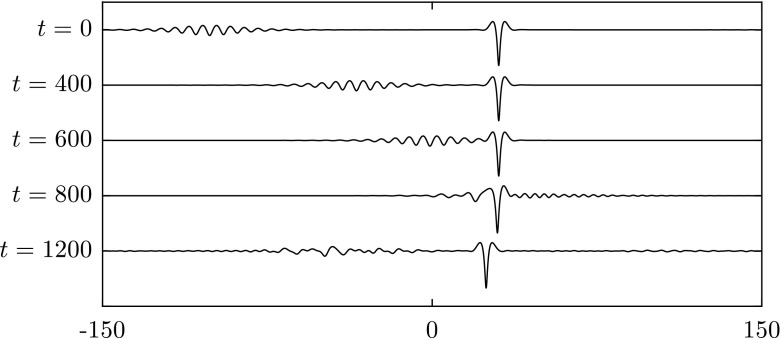
Overtaking collision of two depression waves $c=0.997$ (*left*) and $c=0.83$ (*right*) for $\beta =1$. A frame of reference moving with speed 0.83 is chosen. The wave with small amplitude is suppressed after the interaction

**Fig. 16:**
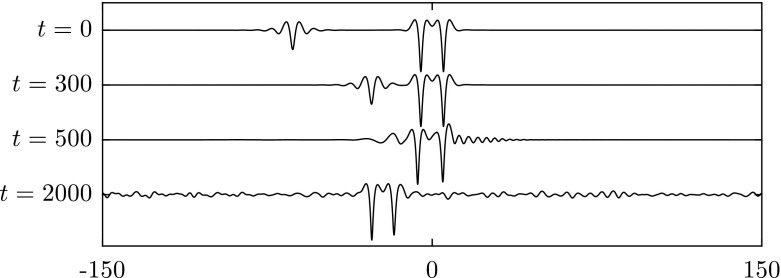
Overtaking collision of a depression wave with $c=0.97$ (*left*) and an elevation wave with $c=0.851$ (*right*) when $\beta =1$. A frame of reference moving with speed 0.851 is chosen. The depression wave is killed off after the interaction

**Fig. 17:**
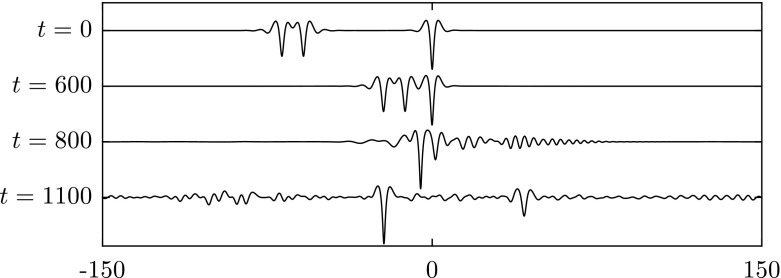
Overtaking collision of an elevation wave with $c=0.9465$ (*left*) and a depression wave with $c=0.87$ (*right*) when $\beta =1$. A frame of reference moving with speed 0.87 is chosen. The elevation wave turns to be a depression one after the interaction

Collision dynamics are investigated further in the results presented in Figs. 15, 16 and 17. These examples are characterised by large differences between the speeds of the chosen waves. In Fig. 15, two depression waves with speed $c=0.997$ and $c=0.87$ are chosen. The results show that wave with smaller amplitude is suppressed after the interaction. A similar outcome is observed in Fig. 16 where a depression and an elevation solitary wave are chosen initially. In Fig. 17, an elevation wave with speed $c=0.9465$ overtakes a depression wave with speed $c=0.87$. After the collision, two depression waves and many small ripples emerge, i.e. the elevation wave evolves into a depression one. The reason for this is that the depression wave is much less energetic than the elevation wave travelling at same wave speed. When energy dispersion is large, the elevation wave cannot maintain its form but transforms into a less energetic wave instead of dispersing out completely. If we switch the roles of the two waves studied above, i.e. a depression wave overtaking an elevation wave, the outcomes are qualitatively similar. Such observations were reported for flexural–gravity wave in [19].

From the numerical experiments of collisions, we have observed that larger waves get larger (and slower) and smaller waves get smaller (and faster). This is due to the inelastic nature of the collisions where energy exchange always takes place.

### Excitation

**Fig. 18:**
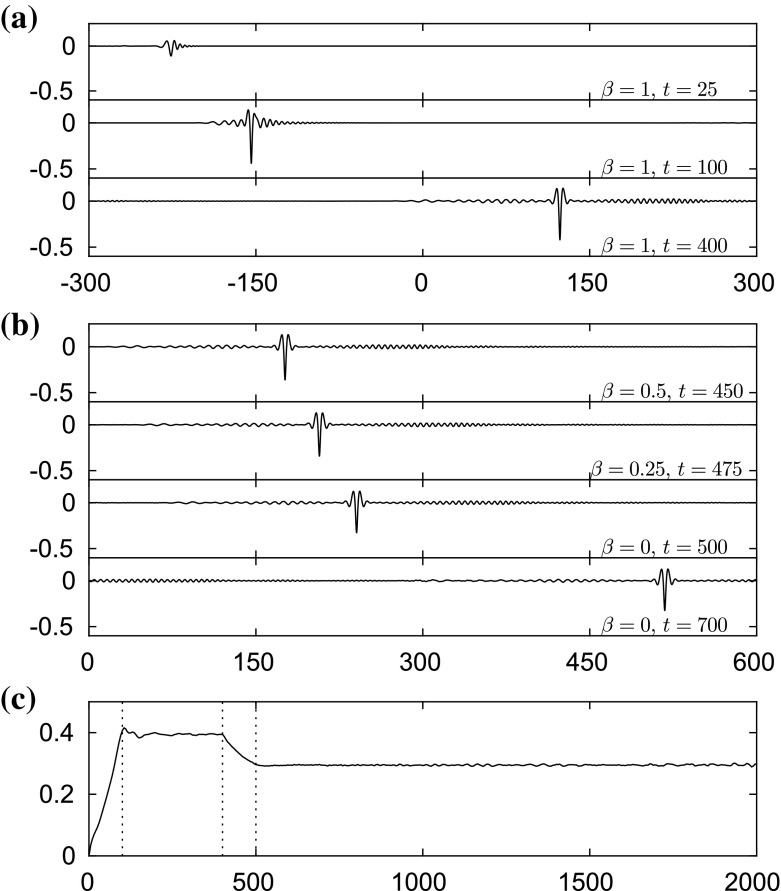
Excitation with a single moving disturbance. It is initially placed and switched on at $x=-250$ with a speed $U=0.99$, and switched off at $t=100$. **a** Snapshots taken at $t=25$, 100, 400 (from *top* to *bottom*). **b** Snapshots taken at $t=450$, 500, 700 (from *top* to *bottom*) where the value of $\beta$ is being reduced during $400\le t \le 500$. The profiles are plotted in [0, 600] instead of $[-300,300]$ for better display by using periodicity. **c** A plot of the maximum slope of the surface versus time *t*. *Vertical dotted lines* are drawn at $t=100$, $t=400$ and $t=500$. All the profiles in (**a**) and (**b**) are sketched in the physical plane

The problem of wave excitation is an important research area that attracts a lot of interest. The minimum of the phase speed is a critical value where the resonance between a moving load, for example, and the surface waves yields interesting phenomena. In this regime, linear theory becomes invalid, and nonlinearity is crucial in describing the physical behaviour of the system. Such types of problem were studied in [14, 15, 19, 20, 25, 26] for flexural–gravity waves and [4] for capillary–gravity waves. In this section, we study this problem in the presence of gravity, surface tension and electric fields. We fix $\beta =1$ and introduce a single moving disturbance $\mathcal {P}$ with a speed $U=0.99$ in the transcritical range (slightly below the minimal phase speed); the disturbance is given by

40 $$\begin{aligned} \mathcal {P}=A\mathrm{e}^{-(x+250-Ut)^2}, \end{aligned}$$

where the amplitude *A* is chosen to be 0.02. The forcing is initially placed at $x=-250$ and later switched off at $t=100$. Snapshots taken at $t=25$, 100, 400 are presented in Fig. 18a. Immediately after removing the pressure disturbance at $t=100$, a depression solitary wave forms on the surface. The long time evolution (up to $t=400$) shows that this depression wave continues to propagate without losing its main structure. Hence, the results confirm the stability of the fully localised response. In the next set of numerical experiments, we reduce the strength of electric field progressively by setting

41 $$\begin{aligned} \beta =1-0.01(t-400),\quad \text {for}~~400\le t \le 500, \end{aligned}$$

and at $t=500$, the electric field is switched off completely by keeping $\beta =0$. During this period, the depression solitary wave maintains its shape and becomes less steep due to energy radiation (see Fig. 18b, c). As the value of $\beta$ decreases to zero, the wave speed rises to approximately 1.385 beyond $t=500$. We continued the numerical computations up to $t=2000$ and found that the depression wave keeps propagating.

**Fig. 19:**
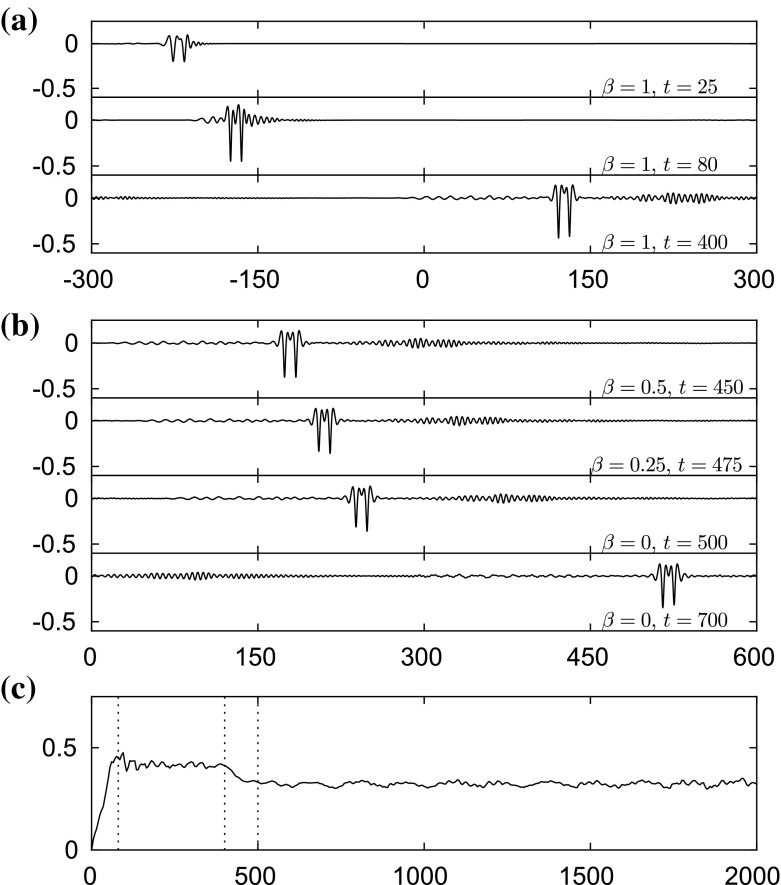
Excitation with two moving disturbances with a distance $d=10$ between them. They are initially placed switched on with a speed $U=0.99$. We switch them off at $t=80$. **a** The snapshots are taken at $t=25$, 80, 400 (from *top* to *bottom*). **b** Snapshots taken at $t=450$, 500, 700 (from *top* to *bottom*) where the value of $\beta$ is being reduced to zero during $400\le t \le 500$. The profiles are plotted (using periodicity) in the interval [0, 600] instead of $[-300,300]$ for better visualisation. **c** A plot of the maximum slope of the surface versus time *t*. *Vertical dotted lines* are drawn at $t=80$, $t=400$ and $t=500$. All the profiles in (**a**) and (**b**) are sketched in the physical plane

The stable elevation wave can be excited by means of two moving disturbances with an appropriate distance between them as in [19] for flexural–gravity waves and in [4] for capillary–gravity waves, for example. We introduce two moving loads

42 $$\begin{aligned}&\mathcal {P}_1=A\mathrm{e}^{-(x+250-Ut)^2}, \qquad \mathcal {P}_2=B\mathrm{e}^{-(x+250-d-Ut)^2}, \end{aligned}$$

where $A=B=0.03$, $U=0.99$ and $d=10$. Both disturbances are switched on at $t=0$ and switched off at $t=80$. It can be seen clearly from Fig. 19 that an elevation wave is generated at $t=80$ and does not disperse out after the removal of the moving loads. This wave also survives while we reduce the value of $\beta$ progressively to zero for $400\le t \le 500$, and maintains its shape afterwards. The stability of this fully localised solution is confirmed by these results.

With the presence of electric fields, excitations can be realised with single or multiple moving pressure disturbances at a relatively low speed, compared to the case of capillary–gravity waves. By further turning down the electric field strength, the excited depression and elevation waves both maintain their wave structure, i.e. capillary–gravity waves emerge. We propose this as an alternative possible way to excite capillary–gravity solitary waves.

## Conclusion

The problem of capillary–gravity flows under normal electric fields was considered. By means of a conformal mapping, we found steady and time-dependent solutions for various values of the dimensionless electric field strength $\beta$. In particular, when $\beta <\beta ^{\dagger }\approx 1.5265$, the properties of waves are qualitatively similar to those for the capillary–gravity waves. The branch of elevation waves split into two separate parts when $\beta >\beta ^{\dagger }$, and the lower part disappears when $\beta >\beta ^{\ddagger }\approx 1.9809$. The stability problem was studied systematically using a time-dependent numerical scheme for $\beta =1$. Several numerical experiments of head-on and overtaking collisions were performed. The stable depression and elevation waves were excited, respectively, using a single load and multiple loads moving with a speed which is slightly less than the minimum phase speed. Overall, the electrified waves have similar properties to those of classical capillary–gravity waves. The main advantage with the presence of normal electric fields is that the waves can be slowed down while keeping the shape. This could open new scenarios for experimentation.
